# Carcinoma of the Breast, the National Screening Programme

**Published:** 1989-05

**Authors:** A. John Webb


### Editorial

### CARCINOMA OF THE BREAST, THE NATIONAL SCREENING PROGRAMME

Broadly speaking we are in a mess over breast carcinoma. It has always been so and anyone industrious enough to study the literature in depth will have realised it. Despite the past twenty years of frenetic investigation, therapeutic revolution and comprehensive trials, the end results are not improved. (Forrest report 1986).

Perhaps the only time when I recall that the whole enterprise was seemingly well appreciated lay during my student and early surgical trainee days—the 1950s. Cooke, Tasker, Jackmann, Milnes Walker, supported by the best of national and international authorities, saw most of these cases fairly advanced. Immediate admission; excision biopsy and frozen section which, if positive was followed by radical mastectomy and irradiation was the vogue. A few small voices stood against this fiercely held dogma, McWhirter in Edinburgh and our own Gordon Paul (almost surreptitiously), performed simple mastectomy coupled with irradiation. Things are somewhat more refined now, but do our results show much benefit?

What is reasonably established fact for this disease; what has been revealed in the interim? Well, for age adjusted breast cancer mortality per 100,000 women (1982–3) England and Wales at 34.5 leads the world! Many cases present earlier than formerly and for many centres, both here and abroad, preoperative diagnosis by fine needle aspiration biopsy (FNAB) is the method of choice and frozen section is infrequently necessary. Apart from a few 'diehards' radical mastectomy is abhorrent and simple mastectomy with some form of axillary 'adventure' is popular. The style of axillary surgery varies enormously from a four node 'scratch out' to a formal tidy block removal of level I and II nodes. Less extensive breast surgery; variously assigned and often loosely termed segmental mastectomy and lumpectomy is practised. Subtotal mastectomy—if one may use the term can suffice for local control until the patient dies of metastases, on the other hand it is realistic to state that its application should involve very careful rules of assessment. Mammography constitutes a great boon for the surgeon and a fascinating extra burden for the radiologist. It is around 90% sensitive for proven breast cancers and can detect preclinical breast cancer. How will it perform in Great Britain within the proposed screening plan? It should come as no surprise to realise that despite the Forrest Report and fervent hopes (Baum 1988, and Rodway 1988) that there are cogent voices of scepticism (Skrabanek 1088, Andersson 1988).

Sadly, under the age of 50, there is uniform agreement that screening is of no benefit in mortality. Over that age it *may* bear fruit. Theoretically it should be beneficial if more '$T_O$, $N_O$' tumours are discovered and this hope has clearly buoyed up so many. Hence the political bandwagon is rolling; the disturbance and organisational ferment will be considerable; the anguish of cost effectiveness will be ever present; we still have to manufacture the surgical sessions and much time will have to be side-tracked into audit, recapitulation, rethink and whatever. In the end we may save 40 deaths in 78,000 women over a ten year period.

On a recent pilgrimage to Canford Cemetery I visited the rose arbour on which the ashes of my late father were scattered. It is usually a lovely bright spring morning and so it was this year. I pass many grave stones, some relate to my former patients and friends. One in particular makes me pause. A lady whose two orphaned sons were friends of my daughters. She succumbed to breast cancer in her late 30s leaving a husband and these two lads. She is just one amongst many and did not deserve her fate so perhaps our successors should not blame us for trying the experiment, for that is what it will be.

A. John Webb

### REFERENCES

Breast Cancer Screening—The Forrest Report 1986, HMSO
BAUM, M. (1988) *Cancer Topics* **7**, 13. Lederle
RODWAY, A. (1988) *Cancer Topics* **7**, 14. Lederle
SKRABANEK, P. (1988) *Lancet* (i) 1155.
ANDERSON, I. *et al. Br. Med. J.* **297**, 943.

